# Autologous micro-fragmented adipose tissue injection provides significant and prolonged clinical improvement in patients with knee osteoarthritis: a case-series study

**DOI:** 10.1186/s40634-023-00668-y

**Published:** 2023-11-16

**Authors:** Arcangelo Russo, Gabriele Cortina, Vincenzo Condello, Marco Collarile, Roberto Orlandi, Riccardo Gianoli, Emanuele Giuliani, Vincenzo Madonna

**Affiliations:** 1 Department of Orthopedics, Joint Prosthetic, Arthroscopic Surgery and Sports Traumatology, Humanitas Castelli, Via Mazzini 11, 24128 Bergamo, Italy; 2 Department of Orthopaedic and Trauma Surgery, University Campus Bio-Medico of Rome, 00128 Rome, Italy; 3 Engineering Department, University of Bergamo, Viale Marconi, 5, 24044 Dalmine, BG Italy

**Keywords:** Micro-fragmented Adipose Tissue (MAT), Mesenchymal Stem Cells or Medicinal Signalling, Cells (MSCs), Knee Osteoarthritis (KOA), Minimal Clinical Important Difference (MCID)

## Abstract

**Purpose:**

Among the conservative strategies to manage patients with symptomatic knee osteoarthritis (OA), an innovative approach exploiting the regenerative capability of adipose tissue and its resident MSCs (Mesenchymal Stem Cells or Medicinal Signalling Cells) has been proposed with encouraging results. This study aims to demonstrate the benefits of autologous micro-fragmented adipose tissue (MAT) injection in the conservative treatment of knee osteoarthritis and whether any variables may affect the outcome. This is a case series single-centre study in which patients underwent intraarticular MAT injection without any associated procedures.

**Methods:**

Based on inclusion and exclusion criteria, 49 patients (67 Knees) were included and retrospectively analysed with a mean follow-up of 34.04 ± 13.62 months (minimum 11 – maximum 59). Patients were assessed through the WOMAC and KOOS questionnaires at baseline (pre-treatment) and 1-, 3-, 6-, 12-, 24- and 36-month follow-up. A minimal clinically important difference (MCID) of at least 7.5 points for the WOMAC pain scale and 7.2 for the WOMAC function scale compared to the baseline value was used.

**Results:**

WOMAC and KOOS scores improved after treatment compared to baseline at all follow-ups with *p* < 0.001. Male gender and Kellgren-Lawrence (KL) grade 2 were associated with smaller improvement in WOMAC and KOOS scores (with respect to females and to KL grade 1, respectively) up to 24 months. The percentage of patients who reach the MCID for WOMAC pain is generally lower than that of patients who reach the MCID for WOMAC function (around 80% at all time points), but it increases significantly over time. Moreover, the baseline score of the WOMAC pain and function influence the outcome. Patients with worse symptoms are more likely to reach the MCID.

**Conclusions:**

Intra-articular knee injection of MAT for the treatment of knee osteoarthritis (KOA), recalcitrant to traditional conservative treatments, proved to be effective in a high percentage of cases. The positive association between a worse pre-operative score and a better clinical response to the treatment would support the idea that intra-articular administration of MAT could be considered in patients with very symptomatic KOA in which joint-replacement surgeries are not indicated (or accepted).

**Level of Evidence:**

IV, case series.

## Background

Among the conservative strategies to manage patients with symptomatic knee osteoarthritis (KOA), intra-articular injections of corticosteroid or hyaluronic acid (HA) have shown satisfactory results in the last decade [1–4]. However, these treatments have not allowed for proven efficacy in changing/reverting the natural history of the disease in many patients [3, 5].

Platelet-rich plasma (PRP) has been advocated as an alternative injective treatment option, given the anti-inflammatory effect of blood growth factors [6, 7]. However, the results are controversial, mainly because of the need for more agreement on the specific PRP formulation and application protocol. Recently, an innovative approach exploiting the regenerative capability of adipose tissue and its resident MSCs (Mesenchymal Stem Cells or Medicinal Signalling Cells) has been proposed with encouraging results [8, 9]. Indeed, extensive in vitro and ex vivo research activity focused on the identification and explanation of the mechanisms of action of MSCs has clearly shown the influence of MSC paracrine activity on reducing inflammation and promoting matrix turnover in osteoarthritis (OA) [10].

Nevertheless, preparing autologous MSCs for injection would require ex vivo culture from a good manufacturing practice facility, which makes the process laborious and expensive [10–12]. Therefore, the availability of a minimally manipulated adipose tissue providing regenerative components in one step is of remarkable clinical relevance and equal effectiveness [13]. Among the available techniques, this research employed a commercial system that provides micro-fragmented and minimally manipulated adipose tissue without expansion or enzymatic treatment [14]. Micro-fragmented adipose tissue (MAT) is obtained through a mild mechanical tissue cluster size reduction in a full immersion closed system. It has already been shown to be safe and promising in different pathologies [12, 15–17]. In particular, the intra-articular injection of MAT for KOA showed early promising results [18–20]. In the literature, several authors have shown how gender, BMI and a higher degree of KOA can influence the clinical response to treatment with MSC [21, 22].

Therefore, the targets of the study are to evaluate If any parameters can influence the response to the treatment with intra-articular injection of MAT for symptomatic KOA; if a good and stable clinical outcome (measured by the Minimal Clinical Important Difference, MCID) is maintained over time and if pre-operative scores of WOMAC and KOOS can influence the MCID.

## Methods

### Study design and patient selection

This is a case series single-centre study conducted over four years. From April 2018 to September 2022, 49 patients (67 knees) underwent autologous MAT intraarticular knee injection in a highly specialised orthopaedic centre (Humanitas Castelli Hospital, Bergamo, Italy). The ethics committee approved the following study (protocol number 35/23 GAV, CET Lombardia 5). Patients’ demographics are presented in Table 1. The inclusion and exclusion criteria are reported in Table 2.

**Table 1:** Patients’ demographics

**N° patients**	**N° knees**		
49	67		
**Males**			
N° (%)	Mean Age	BMI	
28 (57,1)	57,7	26,8 ± 3,5	
**Females**			
N° (%)	Mean Age	BMI	
21 (42,9%)	61,1	27,1 ± 4,6	
**Classification Kellgren Lawrence (% of knees)**			
1°	2°	3°	
31,3%	61,2%	7,5%	
**Follow-up (N° of patients)**			
< 1 year	1—2 years	2—3 years	3—4 years
1	14	7	27

**Table 2:** Inclusion and exclusion criteria

Inclusione criteria	Exclusion criteria
1. MAT intra-articular injection in one or both knees; 2. Age between 18 and 80 years old; 3. Body Mass Index (B.M.I.) < 40; 4. Diagnosis of tibiofemoral osteoarthritis of the target knee/s at Kellgren-Lawrence (KL) grade ≥ 1 based on weight-bearing x-rays performed during the pre-treatment screening; 5. Knee symptoms that have lasted for more than six months; 6. Failure of at least one conservative treatment (activity modification and weight loss, physical therapy, or NSAID); 7. Failure of at least one intra-articular knee injection (corticosteroid, platelet-rich plasma [PRP], or hyaluronic acid [HA]).	1. intraarticular injection or orthopaedic surgical treatment of the lower limbs in the six months before the treatment; 2. valgus/varus deformity ≥ 10°; 3. any clinical condition that could have interfered with the outcome evaluation (i.e. severe hip or ankle OA, a disease of the spine, any severe illness of lower limbs other than knee OA); 4. any other concomitant surgical treatment during the intra-articular injection procedure (arthroscopy, meniscectomy, ligament reconstruction, etc.); any other intra-articular or tendon injection during the same procedure; 5. diagnosis of inflammatory or metabolic disease (rheumatoid arthritis, psoriatic arthritis, ankylosing spondylitis, gout, etc.)

### Clinical assessment and data collection

Patients were evaluated at baseline, during the pre-treatment screening, at 1-, 3-, 6-, 12-, 24-, and 36-months follow-up. Baseline demographics data, BMI, and medical history, including previous conservative and surgical treatments of the target knee/s, were collected. Clinical examination was performed on all the patients. Anteroposterior weight-bearing x-rays of both knees, lateral-lateral, and Merchant view x-rays of the target knee/s were ordered during the pre-surgical screening. In our treatment protocol, the indication for autologous and micro-fragmented adipose tissue intraarticular injection in patients with a KL grade 4 was allowed only upon the patient refused a surgical knee replacement option.

Patients completed WOMAC and Knee injury and Osteoarthritis Outcome Score (KOOS) questionnaires at baseline (pre-treatment) and 1-, 3-, 6-, 12-, 24- and 36-months follow-up. The clinical examination was performed by an investigator not involved in the surgical indication or injection treatment. A minimal clinically important difference (MCID) of at least 7.5 points for the WOMAC pain scale and 7.2 for the WOMAC function scale compared to the baseline value was used, according to Holtz et al. [23]. Furthermore the MCID of at least 10 points for the WOMAC total score compared to the baseline value was used, according to Clement et al. [24].

### Surgical procedure

The adipose tissue harvesting and processing has been previously described [25]. In summary, the lower or the lateral abdomen was chosen as the donor site for adipose tissue harvesting. Before harvesting the fat, the site was injected with an irrigation solution composed of NaCl 0.9% 250 mL, 200 mg/10 ml of mepivacaine 2% (two vials) and 0.5 mg/0.5 mL of adrenaline (1/2 vial). The fat was then harvested using a 13G blunt cannula connected to a Vaclock® 20-ml syringe. The harvested fat was immediately processed in the Lipogems® processing kit (Lipogems International Spa, Milan, Italy). This disposable device progressively reduces the size of the adipose tissue clusters with a mild mechanical action while eliminating oily substances and blood residues with pro-inflammatory properties. The resulting micro-fragmented fat was collected in a 60 ml syringe, positioned for decanting the excess saline solution, and then transferred into several 10 ml syringes to be injected into the patient. Micro-fragmented fat was injected intra-articular in a volume of 10 ml in each knee.

### Post-op rehabilitation protocol

All patients wore an elastic compression band on the harvesting site for 2–3 weeks during the postoperative period. In addition, patients were administered painkillers in the immediate post-op upon request and low molecular weight heparin for ten days. The postoperative protocol was five days of unloading, then full load recovery in the following five days, active and passive motion from the immediate post-op, and proprioceptive exercises from day five post-op.

### Statistical analysis

Analyses were performed using R software v4.1.3 (R Core Team, Vienna, Austria) [26]. Continuous data distribution was assessed by the Shapiro–Wilk test. Parametric or non-parametric tests were performed according to the result of this test. One-Way ANOVA test with Tukey’s post hoc test for pairwise comparison (or Kruskall-Wallis with Dunn’s post hoc test for non-normal data) was used to assess differences among time points or more than two different categories. Student t-test or Mann Whitney U test was used for comparisons between two subgroups. Multilevel linear models were selected based on AIC minimization criteria to evaluate the effect of different variables on the change in clinical outcomes and to adjust estimations for patients treated bilaterally. In addition, analyses were repeated, excluding patients treated bilaterally. *P* values < 0.05 were considered statistically significant.

## Results

### Functional score improvements after treatment

**Fig. 1:**
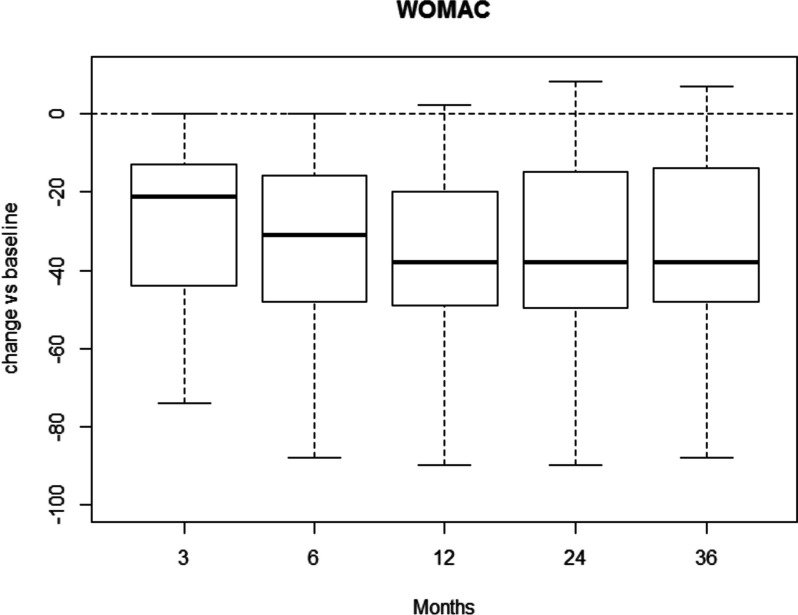
Boxplot of WOMAC score changes compared to baseline (WOMAC at follow-up – WOMAC at baseline, calculated for each patient)

WOMAC score showed improvements after treatment compared to baseline at all follow-ups with *p* < 0.001. Further significant improvements were observed comparing the 3-month WOMAC score with 6- (*p* = 0.016), 12- (*p* = 0.002), and 24-month (*p* = 0.019) evaluations. Figure 1 shows the reduction in WOMAC score compared to the baseline for all time points. Absolute values are reported as well as for each grade of KL classification (Tables 3, 4, 5 and 6).

**Table 3:** Absolute values of each scale of WOMAC score. Data expressed as mean ± standard deviation

	Baseline (*n* = 67)	1-month f.up (*n* = 67)	3-month f.up (*n* = 67)	6-month f.up (*n* = 67)	12-month f.up (*n* = 54)	24-month f.up (*n* = 36)	36-month f.up (*n* = 25)
WOMAC—Pain	10.73 ± 5.24	7.33 ± 4.30	4.63 ± 3.40	3.15 ± 2.94	3.04 ± 3.68	3.64 ± 4.82	4.40 ± 5.40
WOMAC—Stiffness	2.13 ± 2.41	1.70 ± 2.04	1.30 ± 1.87	1.04 ± 1.91	0.96 ± 1.95	0.78 ± 1.51	0.64 ± 1.35
WOMAC—Function	34.76 ± 17.15	23.70 ± 15.12	15.54 ± 11.32	11.21 ± 9.75	10.80 ± 12.41	12.56 ± 15.25	15.36 ± 17.48
WOMAC Total	47.63 ± 22.76	32.73 ± 19.94	21.46 ± 15.41	15.40 ± 13.62	14.80 ± 17.02	16.97 ± 20.55	20.40 ± 23.10

**Table 4:** Absolute values of each scale of WOMAC score in patients with KL 1. Data expressed as mean ± standard deviation

	Baseline (*n* = 36)	1-month f.up (*n* = 36)	3-month f.up (*n* = 36)	6-month f.up (*n* = 36)	12-month f.up (*n* = 32)	24-month f.up (*n* = 25)	36-month f.up (*n* = 19)
WOMAC—Pain	12.03 ± 5.72	8.36 ± 4.86	4.89 ± 4.05	2.58 ± 3.32	2.06 ± 3.51	2.76 ± 4.54	3.63 ± 5.37
WOMAC—Stiffness	2.31 ± 2.44	1.72 ± 1.92	1.25 ± 1.71	0.83 ± 1.73	0.78 ± 1.79	0.48 ± 1.29	0.42 ± 1.22
WOMAC—Function	38.25 ± 18.93	26.42 ± 17.20	15.81 ± 12.85	9.11 ± 10.68	7.38 ± 11.48	9.40 ± 14.56	12.37 ± 17.42
WOMAC	52.58 ± 25.23	36.50 ± 22.59	21.94 ± 17.46	12.53 ± 14.95	10.22 ± 15.92	12.64 ± 19.90	16.42 ± 23.21

**Table 5:** Absolute values of each scale of WOMAC score in patients with KL 2. Data expressed as mean ± standard deviation

	Baseline (*n* = 21)	1-month f.up (*n* = 21)	3-month f.up (*n* = 21)	6-month f.up (*n* = 21)	12-month f.up (*n* = 14)	24-month f.up (*n* = 8)	36-month f.up (*n* = 3)
WOMAC—Pain	8.67 ± 3.58	5.95 ± 2.92	4.24 ± 2.45	3.81 ± 2.06	4.29 ± 2.89	3.88 ± 3.48	3.67 ± 3.21
WOMAC—Stiffness	2.29 ± 2.65	2.14 ± 2.48	1.76 ± 2.34	1.67 ± 2.42	1.64 ± 2.59	1.75 ± 1.98	1.33 ± 1.15
WOMAC—Function	28.05 ± 12.06	19.52 ± 10.90	14.81 ± 9.18	13.90 ± 7.33	16.00 ± 10.94	13.50 ± 7.73	15.00 ± 5.29
WOMAC	39.00 ± 16.12	27.62 ± 15.18	20.81 ± 13.30	19.38 ± 10.97	21.93 ± 15.70	19.12 ± 11.23	20.00 ± 7.55

**Table 6:** Absolute values of each scale of WOMAC score in patients with KL 3. Data expressed as mean ± standard deviation

	Baseline (*n* = 5)	1-month f.up (*n* = 5)	3-month f.up (*n* = 5)	6-month f.up (*n* = 5)	12-month f.up (*n* = 5)	24-month f.up (*n* = 3)	36-month f.up (*n* = 3)
WOMAC—Pain	13.40 ± 2.88	9.40 ± 3.51	6.00 ± 3.00	4.80 ± 3.83	5.60 ± 5.86	10.33 ± 6.35	10.00 ± 5.20
WOMAC—Stiffness	1.00 ± 2.24	0.40 ± 0.89	0.00 ± 0.00	0.00 ± 0.00	0.00 ± 0.00	0.67 ± 1.15	1.33 ± 2.31
WOMAC—Function	43.20 ± 10.89	32.60 ± 12.56	22.00 ± 11.98	17.40 ± 12.50	18.80 ± 18.73	36.33 ± 18.48	34.67 ± 16.17
WOMAC	57.60 ± 12.40	42.40 ± 15.50	28.00 ± 14.95	22.20 ± 16.10	24.40 ± 24.45	47.33 ± 23.67	46.00 ± 19.05

Multilevel linear regression models were used to test the association of different variables to changes in WOMAC score. Male gender was associated with higher WOMAC score (with respect to females) up to 24 months (*p* = 0.053). KL grade 2 was also associated with higher WOMAC scores with respect to KL grade 1, especially at 24 months when this difference was close to statistical significance (*p* = 0.088) (Table 7).

**Table 7:** Factors influencing WOMAC improvements

	**Δ3-month**	**Δ 6-month**	**Δ 12-month**	**Δ 24-month**	**Δ 36-month**
**Intercept** ***P* value**	**-33.2** ** < 0.001**	**-41.6** ** < 0.001**	**-44.2** **0.001**	**-52.4** ** < 0.001**	**-39.2** **0.001**
**Gender (M)** ***P* value**	**14.3** **0.017**	**17.4** **0.011**	**16.3** **0.043**	**21.7** **0.053**	**21.4** **0.124**
**KL = 2** ***P* value**	**-**	**0.54** **0.772**	**0.15** **0.864**	**20.1** **0.088**	**-**
**KL = 3** ***P* value**	**-**	**-11.0** **0.347**	**-3.3** **0.817**	**9.6** **0.683**	**-**
**BMI** ***P* value**	**-**	**-**	**-**	**-**	**1.9** **0.506**
**N (knees)**	**67**	**62**	**51**	**36**	**25**

Δ, difference compared to baseline. Coefficient (i.e. mean changes associated with the index variable) and *p* values are reported. Reference subject: Female, KL grade 1, BMI = 26. Intercept indicated the mean change for a subject with reference values. Values represent absolute adjusted change in score due to 1 unit increase (continuous variable) or absolute variation with respect to the reference category (Gender, KL grade)

*M* male, *KL* Kellgren-Lawrence, *BMI* body mass index

**Fig. 2:**
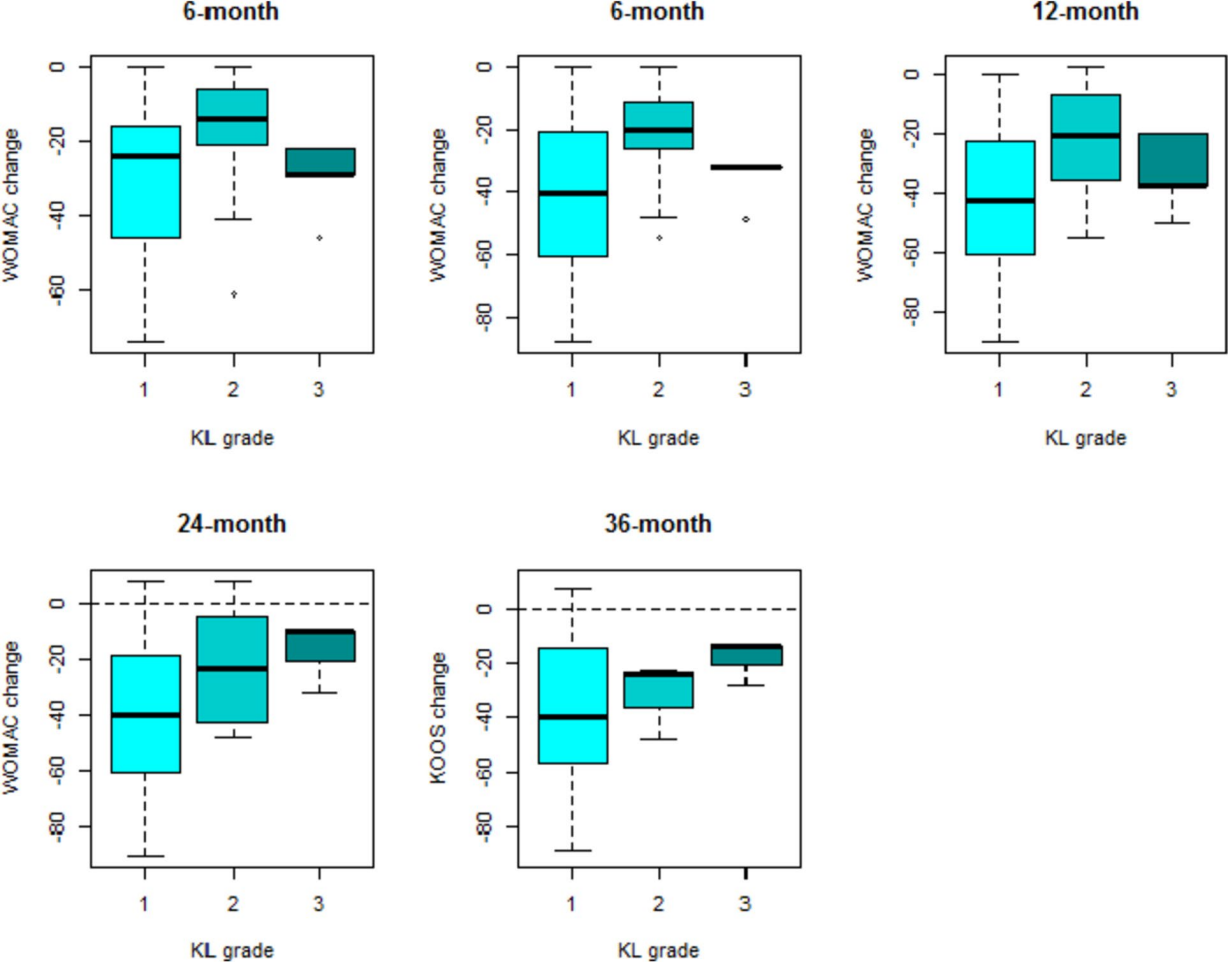
WOMAC change with respect to baseline (WOMAC at follow-up – WOMAC at baseline, calculated for each patient) at all different follow-ups in subjects with KL grade 1, 2 and 3

**Fig. 3:**
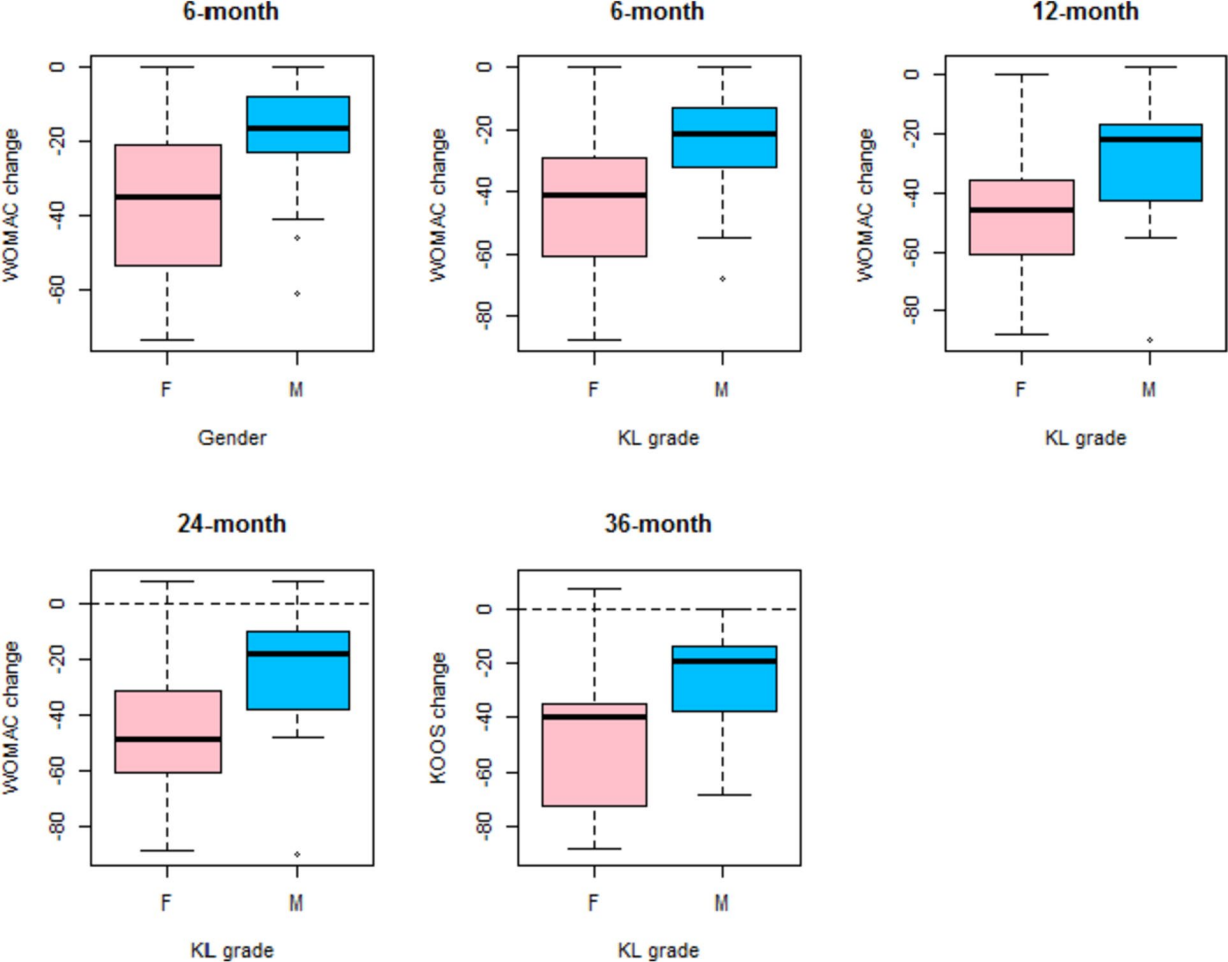
WOMAC change with respect to baseline (WOMAC at follow-up – WOMAC at baseline, calculated for each patient) at all different follow-ups in females (F) and males (M)

Indeed, it was possible to observe that the effect of the treatment decreases for higher KL grades (Fig. 2) and males (Fig. 3) at all different follow-ups.

**Fig. 4:**
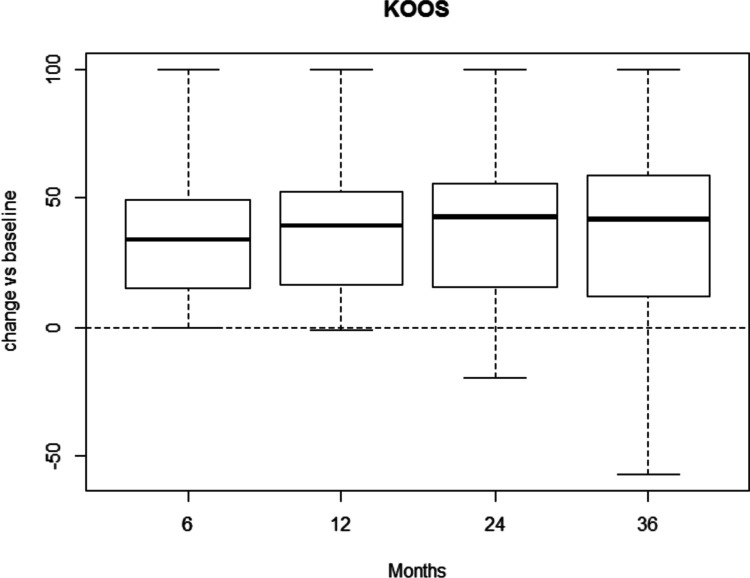
Boxplot of KOOS changes compared to baseline (KOOS at follow up – KOOS at baseline, calculated for each patient)

Similarly, KOOS showed significant improvements at all follow-ups (*p* < 0.001 at 6-, 12-, 24- and 36-months) with respect to baseline. No differences were observed among follow-ups (Fig. 4). Absolute values are reported as well as for each grade of KL classification (Tables 8, 9, 10 and 11).

**Table 8:** Absolute values of each scale of KOOS score. Data expressed as mean ± standard deviation

	Baseline (*n* = 67)	6-month f.up (*n* = 67)	12-month f.up (*n* = 54)	24-month f.up (*n* = 36)	36-month f.up (*n* = 25)
KOOS—Pain	20.81 ± 8.89	7.03 ± 5.23	5.87 ± 6.18	7.25 ± 8.81	8.68 ± 9.72
KOOS—Symptoms	12.22 ± 6.77	4.96 ± 3.99	3.96 ± 4.28	4.36 ± 5.68	5.92 ± 6.58
KOOS—ADL	34.33 ± 17.45	11.19 ± 9.73	10.43 ± 12.21	12.83 ± 15.79	14.64 ± 18.57
KOOS—Sport	16.49 ± 4.47	10.52 ± 5.48	10.50 ± 5.70	9.86 ± 6.40	8.60 ± 6.41
KOOS—QoL	11.73 ± 3.91	5.63 ± 3.68	5.02 ± 4.50	5.36 ± 4.45	5.56 ± 4.86
KOOS Total	38.45 ± 20.39	71.59 ± 14.17	73.61 ± 17.72	73.29 ± 22.26	71.09 ± 25.69

**Table 9:** Absolute values of each scale of KOOS score in patients with KL 1. Data expressed as mean ± standard deviation

	Baseline (*n* = 36)	6-month f.up (*n* = 36)	12-month f.up (*n* = 32)	24-month f.up (*n* = 25)	36-month f.up (*n* = 19)
KOOS—Pain	22.94 ± 9.72	6.03 ± 6.08	4.25 ± 6.29	5.32 ± 8.50	6.95 ± 9.55
KOOS—Symptoms	13.11 ± 7.40	4.08 ± 3.43	2.97 ± 3.59	2.52 ± 4.43	4.58 ± 5.95
KOOS—ADL	37.50 ± 19.63	9.14 ± 10.75	7.22 ± 11.56	9.20 ± 14.68	11.05 ± 17.85
KOOS—Sport	16.92 ± 4.40	10.03 ± 6.44	9.88 ± 6.34	8.80 ± 6.93	7.53 ± 6.73
KOOS—QoL	12.78 ± 3.70	5.89 ± 4.45	4.62 ± 5.09	4.36 ± 4.67	5.00 ± 5.14
KOOS Total	34.46 ± 21.30	73.45 ± 16.43	77.70 ± 17.69	78.29 ± 21.46	75.84 ± 25.81

**Table 10:** Absolute values of each scale of KOOS score in patients with KL 2. Data expressed as mean ± standard deviation

	Baseline (*n* = 21)	6-month f.up (*n* = 21)	12-month f.up (*n* = 14)	24-month f.up (*n* = 8)	36-month f.up (*n* = 3)
KOOS—Pain	17.71 ± 5.75	8.14 ± 3.58	7.21 ± 4.02	8.50 ± 5.83	7.33 ± 2.52
KOOS—Symptoms	11.19 ± 5.39	6.71 ± 4.26	5.00 ± 3.46	6.12 ± 2.95	3.67 ± 1.53
KOOS—ADL	28.05 ± 12.06	13.90 ± 7.33	14.71 ± 9.79	15.12 ± 10.08	14.67 ± 5.69
KOOS—Sport	15.48 ± 4.59	11.14 ± 4.26	11.14 ± 4.85	12.12 ± 5.06	10.67 ± 5.77
KOOS—QoL	9.86 ± 3.29	5.71 ± 2.76	6.21 ± 3.49	7.12 ± 2.80	4.67 ± 2.89
KOOS Total	45.26 ± 16.25	68.31 ± 11.00	67.50 ± 16.08	68.89 ± 15.75	72.49 ± 7.39

**Table 11:** Absolute values of each scale of KOOS score in patients with KL 3. Data expressed as mean ± standard deviation

	Baseline (*n* = 5)	6-month f.up (*n* = 5)	12-month f.up (*n* = 5)	24-month f.up (*n* = 3)	36-month f.up (*n* = 3)
KOOS—Pain	24.60 ± 4.51	11.00 ± 4.85	12.40 ± 8.02	20.00 ± 8.66	21.00 ± 6.93
KOOS—Symptoms	15.80 ± 6.53	7.00 ± 4.64	8.00 ± 8.22	15.00 ± 8.66	16.67 ± 0.58
KOOS—ADL	42.80 ± 10.35	17.00 ± 12.00	19.20 ± 19.27	37.00 ± 19.05	37.33 ± 18.48
KOOS—Sport	18.40 ± 2.19	10.60 ± 1.52	11.60 ± 0.89	12.67 ± 2.31	13.33 ± 1.15
KOOS—QoL	14.60 ± 1.52	5.60 ± 1.52	4.40 ± 4.51	9.00 ± 3.46	10.00 ± 1.73
KOOS Total	25.81 ± 6.29	66.29 ± 9.23	64.65 ± 21.43	43.38 ± 23.24	39.61 ± 13.01

As per WOMAC, changes in KOOS were associated with gender and KL grade. In particular, males showed a lower mean increase compared to females (ranging from -13.3 to 19.4 points, depending on the different follow-ups), even if only at 6 and 12 months, this difference was significant. KL grade 2 was associated with lower improvement up to -26.2 points at 24-month follow-up compared to subjects with KL grade 1 (*p* = 0.042) (Table 12).

**Table 12:** Factors influencing KOOS improvements

	**Δ 6-month**	**Δ 12-month**	**Δ 24-month**	**Δ 36-month**
**Intercept** ***P* value**	**42.2** ** < 0.001**	**48.9** ** < 0.001**	**56.1** ** < 0.001**	**43.6** ** < 0.001**
**Gender (M)** ***P* value**	**-13.3** **0.033**	**-14.8** **0.042**	**-18.7** **0.113**	**-19.4** **0.158**
**KL = 2** ***P* value**	**-4.9** **0.341**	**-10.9** **0.085**	**-26.2** **0.042**	**-**
**KL = 3** ***P* value**	** + 9.6** **0.418**	** + 2.0** **0.881**	**-9.5** **0.701**	**-**
**N (knees)**	**62**	**51**	**36**	**25**

Δ, difference compared to baseline. Coefficient (i.e. mean changes associated with the index variable) and *p* values are reported. Reference subject: Female, KL grade 1. Intercept indicated the mean change for a reference subject. Values represent absolute adjusted change in score due to 1 unit increase (continuous variable) or absolute variation with respect to the reference category (Gender, KL grade)

*M* male, *KL* Kellgren-Lawrence

**Fig. 5:**
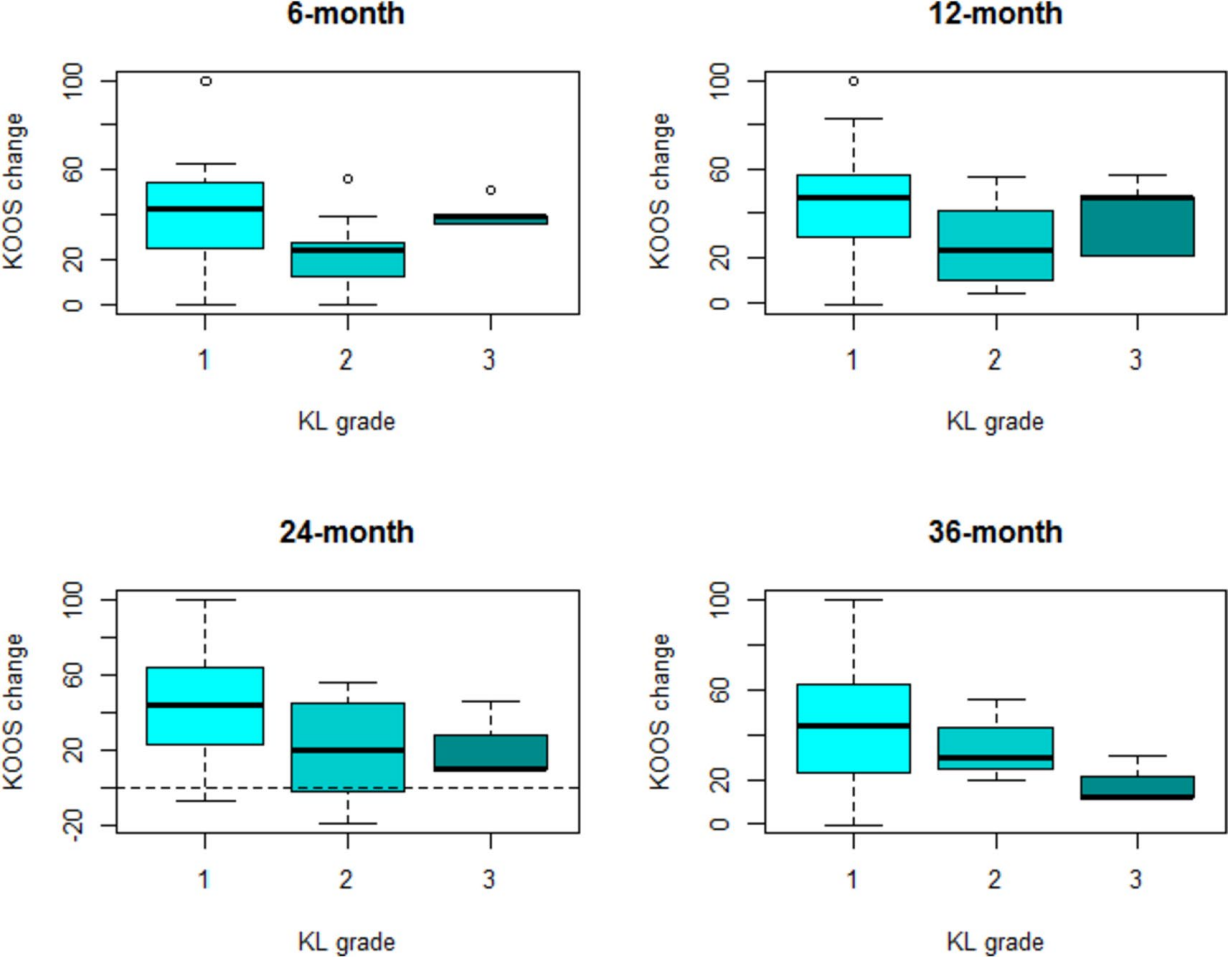
KOOS change with respect to baseline (KOOS at follow-up – KOOS at baseline, calculated for each patient) at all different follow-ups in subjects with KL grade 1, 2 and 3

**Fig. 6:**
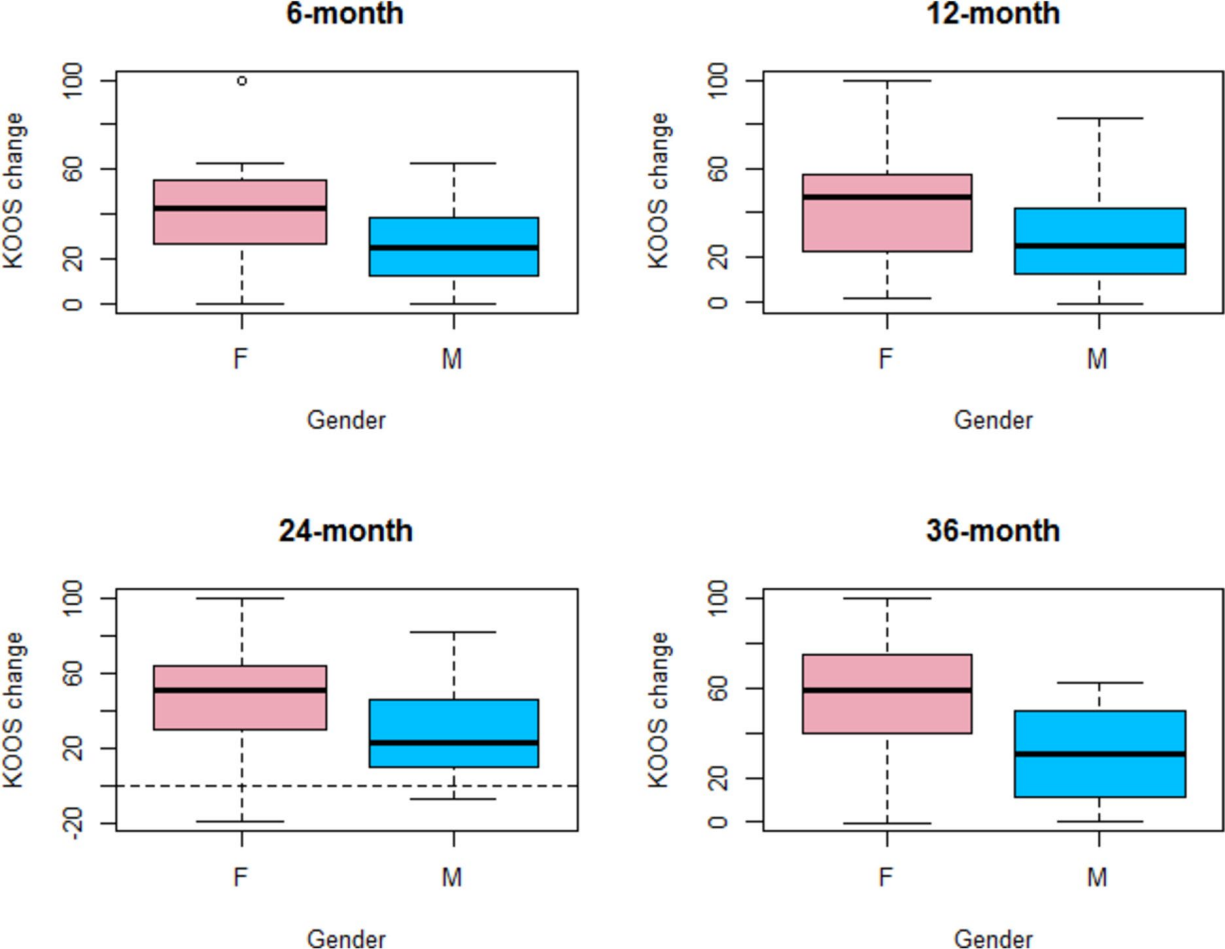
KOOS change with respect to baseline (KOOS at follow-up – WOMAC at baseline, calculated for each patient) at all different follow-ups in females (F) and males (M)

It was possible to observe reduced improvement for patients with KL grade 2 compared to KL 1 (Fig. 5), as well as depending on gender (Fig. 6).

### Functional score and Minimal Clinically Important Difference (MCID)

#### WOMAC pain, function and total score

The percentage of patients who reach the MCID for WOMAC pain is generally lower than that of patients who reach the MCID for WOMAC function, but it increases significantly over time. Otherwise, the percentage of patients reaching the MCID for WOMAC function remains around 80% at all time points. The same was observed for WOMAC total score (Table 13).

**Table 13:** Compared to follow-up, the percentage of patients with scores above the MCID for WOMAC pain and WOMAC function and WOMAC total score

Follow up	N° of patients	% patients > MCID for WOMAC pain	% patients > MCID for WOMAC function	WOMAC total score
3 months	67	28.3%	76.1%	76.1%
6 months	67	41.8%	79.1%	80.6%
12 months	54	46.3%	81.5%	79.6%
24 months	36	50.0%	83.3%	77.8%
36 months	25	52.0%	80.0%	84.0%

#### Influence of baseline values on MCID in WOMAC pain, function and total score

Baseline WOMAC pain values are significantly different between those who achieve the MCID and those who do not attain it at different times (excluding 36 months) (Table 14).

**Table 14:** Influence of baseline values on MCID in WOMAC pain, data are reported as the median (interquartile range)

Follow up	N° of patients	< MCID	> MCID	*P* value
3 months	67	40 (22, 51.25)	67 (62.5, 80)	< 0.001
6 months	67	34 (21, 48)	65 (47, 80)	< 0.001
12 months	54	38 (24, 52)	61 (47, 80)	< 0.001
24 months	36	43 (17.75, 58.75)	69 (46.5, 81)	0.008
36 months	25	47 (23.25, 67.25)	56 (40, 88)	0.156

Similarly, the WOMAC function values at baseline are also significantly different between those who achieve the MCID and those who do not at different times (excluding 36 months) (Table 15). The same was observed for WOMAC total score (Table 16).

**Table 15:** Influence of baseline values on MCID in WOMAC function, data are reported as the median (interquartile range)

Follow up	N° of patients	< MCID	> MCID	*P* value
3 months	67	21 (15, 29)	52 (40, 67)	< 0.001
6 months	67	21 (15, 24.75)	52 (40, 67)	< 0.001
12 months	54	19.5 (11.2, 37.8)	51.5 (40, 68)	0.002
24 months	36	15 (10.5, 53.2)	52 (42, 79.2)	0.034
36 months	25	26 (9, 66)	52 (41.5, 72.5)	0.134

**Table 16:** Compared to follow-up, the percentage of patients with scores above the MCID for WOMAC pain and WOMAC function and WOMAC total score. data are reported as the median (interquartile range)

Follow up	N° of patients	% patients > MCID for WOMAC total score	*P* value
3 months	67	52 (40, 67)	< 0.001
6 months	67	51.5 (40, 66.8)	< 0.001
12 months	54	52 (40, 69)	< 0.001
24 months	36	51.5 (42, 80)	0.008
36 months	25	52 (42, 71)	0.156

#### KOOS pain, symptoms, ADL, sport, QOL

At 6 and 12 months, the percentages of subjects who reach the MCID in the various subscales are shown in the table (Table 17).

**Table 17:** Compared to follow-up, the percentage of patients with scores above the MCID for KOOS subscales. Data are reported as the median (interquartile range)

Follow up	N° of knee	% patients > MCID for KOOS pain	% patients > MCID for KOOS Symptoms	% patients > MCID for KOOS ADL	% patients > MCID for KOOS Sport	% patients > MCID for KOOS QoL
6 months	67	28.5 (20.0, 39.6) *p* value < 0.001	20.5 (14.3, 30.6) *P* value < 0.001	30.3 (20.4, 43.8) *P* value < 0.001	14.4 (9.7, 21.4) *P* value < 0.001	27.9 (22.8, 34.4) *P* value = 0.072
12 months	54	30.9 (20.2, 41.2) *P* value = 0.005	24.2 (12.2, 38.6) *P* value < 0.001	31.2 (20.2, 43.2) *P* value < 0.001	13.4 (1.9, 29.4) *P* value = 0.059	32.5 (24.1, 38.6) *P* value = 0.120

#### Influence of baseline values and follow-up on MCID in KOOS subscales

For Pain, Symptoms, ADL and Sports subscales (even if only at 6 months), a statistically significant difference between the baseline values of KOOS is noted between those who reach the MCID and those who do not reach it, with worse baseline values for those who reach it (Table 18).

**Table 18:** Compared to follow-up, the percentage of patients with scores above the MCID for KOOS subscales Pain, Symptoms, ADL and Sports subscales

Follow up	*N* =	Pain	Symptoms	ADL	Sport	QoL
6 mesi	67	64.2%	40.3%	74.6%	11.9%	11.9%
12 mesi	54	66.7%	42.6%	79.6%	7.4%	31.5%

### Complications

The most common complication in this study was knee swelling and pain, occurring in 7 (10,4%) knees. Moreover, three patients (6%) reported some ecchymosis on their abdomen which was self-limited. There can be considered mild adverse events after this type of procedure. All of these cases were treated with cryotherapy, common analgesics and rest for a few days. No severe adverse event was recorded. At the final follow-up, no patients underwent TKA.

## Discussion

The main finding of this study was that the intra-articular knee injection of micro-fragmented adipose tissue (MAT) allowed for a significant and stable improvement of all the clinical outcomes at 24 months follow-up. Moreover, this study showed that the male gender and a higher degree of KL determine a more contained clinical response. These results align with Gobbi et al., which showed that the male gender and a higher degree of KL are associated with reduced improvement after MAT injection. The same author has shown that ageing can also affect the clinical response. The present study did not show this correlation, but it must be kept in mind that the mean age of the enrolled patients (59.4) is lower than that of Gobbi et al. (70.7) [21]. Data from this study agree with those reported by a recent meta-analysis by Kim et al. that showed significant pain relief at 6 months and 12 months and functional improvement at 6 months and 12 months post-injection [11]. In addition, the stable improvement in KOOS and WOMAC scale in our cohort is similar to those of Roato et al., that showed improvement in WOMAC and VAS after 18 months of follow-up [27] and to those of Spasovski et al. [28] and Hudetz et al. [18], as well. Furthermore, no severe adverse event was recorded, confirming the safety profile already demonstrated [16, 17, 20, 29]. Nevertheless, caution in using this treatment is still recommended, especially since mild adverse events (knee pain, joint swelling, and injection site pain) can occur in the first weeks after treatment. Therefore it is essential to educate the patient [30].

The use of adipose tissue as a source of regenerative cells has increased over the last few years. This is because adipose tissue offers several advantages over other cell sources, such as bone marrow, including minimal invasive harvesting and a higher yield of regenerative cells. Moreover, mesenchymal stem cells from adipose tissue have been demonstrated to possess higher immunomodulatory and thropic activity than bone marrow cells [31–33].

Microfragmentation of adipose tissue is a convenient and safe way to exploit its regenerative capability in a one-step technique. In addition, the MAT used in this study has been characterised by an intact stromal vascular niche rich in mesenchymal cells [34], and some authors supported the idea that these peculiar features can increase the treatment efficiency compared to other methods [35, 36].

In this study a total of 49 subjects (67 knees) were enrolled with a mean follow-up of 34.04 ± 13.62 months. All scores improve significantly from 3 months after treatment, continue to improve up to 6 months (*n* = 67), and then remain stable for up to 24 months. During the follow-up, no patient underwent prosthetic surgery.

Regarding the therapeutic effect of MAT injections on the achievement of MCID, Garza et al. [37] showed that 62% of their treatment group had a WOMAC score above MCID at 6 months. In contrast, Freitag et al. [38] showed that 94.4% of their treatment group were above MCID at 12 months. However, expanded adipose tissue MSCs were used in this cohort. Recently, Zaffagnini et al. showed how a single MAT injection was not superior to PRP injection; moreover, both MAT and PRP provided significant and similar clinical improvement up to 24 months of follow-up. The radiographic evaluation with the KL classification did not show any worsening in KOA severity at the final follow-up for both treatment groups [39]. Gobbi et al. performed a Randomized Clinical Trial comparing MAT with leukocyte‑poor platelet‑rich plasma plus hyaluronic acid with a 2 years follow-up. They showed that both treatments lead to significant clinical improvement without a relevant difference between each other within a cohort of patients with KL grades 1–2 [40] with an improvement in KOOS score in line with the results of this research.

In the present study, the percentage of patients who reach the MCID for WOMAC pain is generally lower than that of patients who reach the MCID for WOMAC function (around 80% at all time points), but it increases significantly over time. These steady results were also reported by Cattaneo et al. [16], although their patients received an arthroscopic surgery in addition to MAT injection and are in line with what emerged from a recent meta-analysis that showed a steady with a slight decrease in WOMAC score after 24 months [41].

Regarding KOOS scores, the results of the present study are in line with those of Boffa et al., which showed that PRP injections for KOA provide stable responsiveness at 6 and 12 months follow-up with encouraging results in terms of MCID (> 80% of patients at each follow-up) [42]. Moreover, the authors highlighted how the female sex is associated with a better clinical response, confirming what emerged from the present study.

The study also highlighted how the MCID is influenced by the baseline score of the WOMAC; in fact, patients with worse symptoms are more likely to improve. This evidence has already been highlighted by Schiavone Panni et al. about the VAS scale [43]. A possible interpretation relies on the fact that a worse baseline score condition might reflect a higher inflammatory status of the joint. In fact, it is known that adipose-derived MSCs are very responsive to inflammation [44–46], higher levels of inflammatory mediators might activate more the pro-regenerative activity of MAT, eventually determining a higher clinical outcome. These observations were emphasised by Heidari et al., that showed highly statistically significant improvement in clinical outcome and quality of life at 2 years follow-up in patients with a bad pre-operative OKS score [22]. In addition, some initial findings have demonstrated an efficient response of chondrocytes and proteoglycan synthesis following MAT injection, as observed by Boric et al., which used functional MRI to assess glycosaminoglycan content in hyaline cartilage. At 24 months follow-up, they showed a significant increase in the glycosaminoglycan content, suggesting the positive effects of MAT injections [47]. This might challenge the natural course of ageing and OA processes, including the loss of proteoglycans in the extracellular matrix [48]. If confirmed, this would have enormous implications since other conservative or surgical treatments have never shown precise results that support their capacity to modify the natural history of the disease.

These results allow us to consider adipose tissue-derived MSCs as a possible second-line injective therapeutic alternative for a large population cohort affected by OA, with apparently better results as emerged from the study of Dallo et al., from which it emerges that adipose tissue-derived MSCs showed better clinical results in Tegner and KOOS symptoms at six months and Tegner at 12 months than leucocyte-poor platelet-rich plasma (LP-PRP) plus hyaluronic acid (HA) [49]. Indeed, this study is only based on clinical findings; therefore, the subjective patient perception could have also had a relevant role. However, in a previous study, the authors evaluated objective data such as the N-glycan profile in synovial fluid by UPLC analysis and glycosaminoglycan content in articular cartilage by dGEMRIC (delayed gadolinium-enhanced magnetic resonance imaging of cartilage)-enhanced MRI. While the first analysis failed to find any possible difference between values at baseline and at 12-month follow, the dGEMRIC index showed an improvement in 53% of the patients and a worsening in 15% of them after autologous microfragmented adipose tissue injection. This would suggest a different response of patients to the treatment, although the lack of clinical findings for these patients, except for the VAS score, does not allow for any direct correlation between subjective satisfaction and imaging results [48].

The present study has several limitations, including the small number of patients and a wide range of follow-ups. Another limitation was the assessment of clinical outcomes only, without any imaging or biochemical evaluation of the possible effect of the treatment on cartilage tissue. The lack of a control group exposes the data of the present study to a possible bias linked to the placebo effect, well highlighted and documented by the recent meta-analysis by Previtali et al. [50]. Moreover, the lack of a control group could overestimate the results of the present study, in which emerged a great clinical response in patients with lower scores. A good clinical response could also be justified by the use of painkillers or as a result of proprioceptive exercises that each patient is advised to undergo after MAT injection.

At the same time, it is of great importance that some patients who reached a follow-up of 40 months maintained an improvement in clinical outcomes without resorting to surgical procedures. The presence of several patients treated bilaterally may be considered a limitation of the study. Indeed, analyses performed excluding these patients suggested a more relevant role for continuous variables (age, in particular), rather than categorical (gender and KL grade), possibly due to the lower number of subjects included in each category. Nevertheless, the statistical approach used in the study, i.e., multilevel modelling, allows accounting for the correlation of data deriving from bilateral patients and provides net estimation of the effects of all variables whilst avoiding excluding patients from the analysis.

A strength of this study is that it complied with almost all the points of the MIBO guidelines checklist [51] and the absence of surgical procedures, such as arthroscopic procedures associated with adipose tissue-derived MSCs. Compared to previous works by the same author [17, 20], this study highlighted the potential of adipose tissue-derived MSCs without confounding factors that could alter the clinical scores.

## Conclusions

Intra-articular knee injection of MAT represents an effective and safe second-line injective treatment for KOA recalcitrant to traditional conservative treatments, with a stable and prolonged effect. Furthermore, it can be considered in patients with moderate to severe knee symptoms based on the positive association between a worse pre-operative score and a better clinical response or in which joint-replacement surgeries are not indicated (or accepted). However, to establish if this treatment may be proposed as an early approach to knee OA, more specific studies, possibly with longer follow-up, are needed to analyse the biological impact of intra-articular injection of MAT on the natural history of the disease.
